# Behavior profile of children with nephrotic syndrome

**DOI:** 10.4103/0019-5545.49452

**Published:** 2009

**Authors:** Prathama Guha, Arun De, Malay Ghosal

**Affiliations:** Department of Psychiatry, Calcutta Medical College, 88 College Street, Kolkata - 700 073, West Bengal, India; 1 Department of Pediatrics, Calcutta Medical College, 88 College Street, Kolkata - 700 073, West Bengal, India

**Keywords:** Behavior disturbance, nephrotic syndrome, psychiatric profile

## Abstract

**Background:**

Nephrotic syndrome, a primarily paediatric disease, is associated with a high relapse rate. Studies have reported behavioral and psychological difficulties in children with nephrotic syndrome, their caregivers and siblings, a factor that is likely to influence the overall outcome of the disease in an adverse manner. In clinical practice, however, the psychosocial aspects of care may be overlooked in the pressure to treat the disease process, unless their importance is stressed by appropriate evidence.

**Objectives:**

The study aims to assess the prevalence of behavior abnormalities in children with nephrotic syndrome attending the renal clinic of a state medical college in eastern India and to compare this with the prevalence in a control group of school children without any detectable physical illness. It also aims to explore the relationship between sociodemographic, disease, and treatment related variables and behavioral abnormalities in the nephrotic syndrome group.

**Materials and Methods:**

We assessed the prevalence of behavior abnormalities in 50 consecutive children with nephrotic syndrome attending the renal clinic of a state medical college and 51 school children as controls using the Developmental Psychopathology Checklist (DPCL). We also assessed the statistical association between sociodemographic, disease and treatment related variables and behavior profile in the nephrotic children group.

**Results:**

Prevalence of behavior disturbance in children with nephrotic syndrome was 68%, significantly higher than that in the control group (21.6%). The behavior abnormalities found in the nephrotic syndrome group were hyperkinesis, obsessive compulsive neurosis, conduct disorder, and emotional disorder, in that order. Frequency of relapse and low socioeconomic status showed significant association with presence of behavior disturbance in the nephrotic syndrome group. This association persisted even after adjusting for other sociodemographic, disease, and treatment related variables, including steroid therapy.

## INTRODUCTION

Nephrotic syndrome is primarily a paediatric disease, its prevalence in children being 15 times greater than in adults. A vast majority of children with nephrotic syndrome suffer from the relatively benign steroid sensitive minimal change disease. The relapse rate, however, continues to be high, and the chances of relapse after a first episode is still as high as 30 to 40%.[1] Any chronic physical illness, especially in children, has biological, behavioral, and social manifestations that have implications for the mental health, social and personality development of the child, and family coping.[2] This would naturally apply to children with nephrotic syndrome too, because of its long drawn relapsing and remitting course.[3,4] Prolonged corticosteroid treatment may also contribute to behavioral disturbances in this especially vulnerable population. However, in busy clinical practice the psychosocial aspect of care may be overlooked in the pressure to treat and control disease processes, unless their importance is stressed by appropriate evidence.[2,5,6]

There have been a few studies in India and abroad documenting the behavioral difficulties in children suffering from nephrotic syndrome,[7–9] but these need to be replicated in clinic as well as community based populations. Our study aims to measure the prevalence of behavioral problems in children with nephrotic syndrome attending a special clinic at a state medical college. This is compared to the prevalence of behavioral problems in a control group of school children with no detectable physical illness. It also aims to look into the association, if any, between behavioral problems and sociodemographic as well as disease related factors, including use of corticosteroids.

## MATERIALS AND METHODS

### Sample selection

All consecutive patients between 5 and 15 years, attending the renal clinic of the Calcutta Medical College and Hospital, Kolkata, Eastern India, were included in the study, provided they fulfilled our inclusion and exclusion criteria. The inclusion criteria were: a) Children with a diagnosis of nephrotic syndrome; b) Those aged between 5 and 15 years; and c) Both children and guardians capable of understanding instructions in the local dialect. The exclusion criteria included: a) Duration of illness less than six months; b) Presence of mental retardation; and c) Presence of any other medical or surgical illness. A standard Indian Council for Medical Research form was used to obtain informed consent from the children's parents or legal guardians.[10] Wherever possible, depending on the child's capabilities, we obtained assent from the child as well.

Children above 15 years were not included in the study as idiopathic nephrotic syndrome is overwhelmingly a disease of children and most studies on nephrotic syndrome done to date have focused on the paediatric age group.[3,4] Besides, the developmental psychopathology checklist (DPCL) which was used as an important assessment tool in the study has been validated in children *below* 16 years. The reason for choosing five years as the lower age limit was that parents of children less than five years were not very cooperative for detailed assessment. Besides, it was difficult to assess psychopathology at a very young age, when age appropriate behavioral variations were often construed as pathological behavior by children's parents. In addition, in the original sample tested during the development of the DPCL, the lowest mean age of children with significant symptomatology was 5.3 years.

Normative data was collected by one of the investigators from a suburban school. Initially a list of all students studying from standard I to standard X was drawn up from the attendance registers. Using a computer generated random number list, a total of 55 children were selected. Those having any physical illness were excluded after consultation with the class teacher and parent(s) and clinical examination. Fifty one children were finally selected as controls. After obtaining informed consent, an interview was sought of the children and their parents, and the DPCL was administered to assess the presence of behavioral problems.

### Study design

The behavioral profile of the children were assessed by a psychiatrist using the DPCL, a reliable and valid instrument for assessing psychopathology in Indian children in clinical settings. This tool was developed at the National Institute of Mental Health and Neurosciences, Bangalore by Kapur and colleagues in 1994.[11] The DPCL is based on a dimensional rather than categorical approach. It identifies the following clusters of developmental problems/disorders: emotional disorders, hyperkinesis, childhood psychosis, learning disorder, hysterical syndrome, conduct disorder, autism, and obsessive compulsive neurosis.

Assessment by DPCL was followed by independent clinical examination by two psychiatrists (Prathama Guha and Malay Ghosal). This was done to supplement the diagnoses made by DPCL. The ICD – 10 Classification of Mental and Behavioral Disorders was followed by the psychiatrists. Diagnosis corresponding to each cluster of DPCL were as follows – Emotional disorders (emotional disorders with onset specific to childhood), hyperkinesis (hyperkinetic disorders), childhood psychosis (schizophrenia, acute and transient psychotic disorders, other non-organic psychotic disorders), learning disorders (specific developmental disorders of scholastic skills), hysterical syndrome (dissociative/somatoform disorders), conduct disorder (conduct disorder), autism (pervasive developmental disorders), and obsessive compulsive neurosis (obsessive compulsive disorder).

The psychiatrists were blind to the children's clinical and sociodemographic status. The treating paediatrician, who was in turn was blind to the patients’ psychiatric status, recorded all necessary clinical and sociodemographic details. These were documented in standard case record forms and follow-up data sheets. To assess the socioeconomic status the Kuppuswamy scale was used. This instrument has been used in various studies in India and has good reliability and validity.[12]

As for collection of control data, care was taken to obtain informed consent from the parents of school children. The DPCL was used to record data after interview with the parents and children. Sociodemographic data were collected using the same proforma as in patients with nephrotic syndrome.

### Data analysis

The data was analyzed using the SPSS 12.00 for windows (SPSS Inc., Chicago, USA). Descriptive statistics like frequency, mean, standard deviation were used to describe the data. The Fisher's exact test was done to compare the prevalence of behavioral abnormalities in the nephrotic syndrome children and the control group. Logistic regression analysis was performed to measure the association between sociodemographic and clinical variables and behavioral abnormality in the nephrotic syndrome group.

## RESULTS

The behavioral profile of 50 patients of nephrotic syndrome were compared with that of 51 age and sex matched controls.

Out of 50 patients, 33 (66%) were male and 17 (34%) female. Their mean age was 8.3 years (range 5-15 years). Thirty six children (72%) hailed from a rural background, with the remaining 14 (28%) coming from urban areas. Thirty percent belonged to the lower, 54% upper lower, and 16% lower middle socioeconomic status. Mean age of maternal education was 4.5 years.

The mean age of onset of disease, as calculated from retrospective records or interview with informants, was 4.17 years (range 4-11 years). All patients belonged to the steroid responsive variety, and 33 out of 50 patients were receiving corticosteroids at the time of assessment. Majority of the patients were on alternate day steroid therapy (1.5 mg/kg every alternate day).

In the control group, 60% were male, with a mean age of 8.19 years (range 5-15 years). Sixty six percent came from a rural background. Twenty two hailed from the lower, 58% from upper lower, and 20% from a lower middle socioeconomic status. The mean years of maternal education were 4.66 years [Table 1].

**Table 1:** Sociodemographic profile of nephrotic syndrome and control group

Nephrotic syndrome	Control
Male (*n*) 33	31
Female (*n*) 17	20
Mean age (yrs) 8.3	8.19
Rural (*n*) 36	34
Urban (*n*) 14	17
Low SES (*n*) 42	39
Mean maternal education (yrs) 4.5	4.66

SES = Socioeconomic status

Using appropriate statistical methods (Chi square tests for sex, rural urban divide and upper or lower socioeconomic status; and *t*-test for age) the two samples (nephrotic syndrome and control) were found to be comparable.

In the nephrotic syndrome group, presence of behavioral disturbance in any of the subscales of DPCL was recorded in 34 (68%) children. Similar disturbances were documented in 11 children (21.6%) of the control group. Applying Fisher's exact test, this difference was statistically significant (*P*=0.01).

In the nephrotic syndrome group, 16 patients suffered from hyperkinesis (32%), four had conduct problems (8%), three learning difficulties (6%), and eleven scored positive in the obsessive compulsive neurosis subscale (22%).

In comparison, six children in the control group scored positive in the hyperkinesis subscale (11.8%), one in the emotional disorders subgroup (2%), and four suffered from symptoms of obsessive compulsive neurosis (7.8%) [Table 2].

**Table 2:** Behavioral profile of children with nephrotic syndrome and control group

	Nephrotic syndrome (%)	Control (%)
Hyperkinesis (*n*)	16 (32)	06 (11.8)
Conduct disorder (*n*)	04 (8)	00
Learning disorder (*n*)	03 (6)	00
Emotional disorder (*n*)	00	01 (2)
O-C neurosis (*n*)	11 (22)	04 (7.8)
Psychosis (*n*)	00	00
No behavioral abnormality (*n*)	16 (32)	40 (78.4)
Total (*n*)	50 (100)	51 (100)

The effects of various sociodemographic and clinical variables on the behavioral outcome were looked into. In children with nephrotic syndrome, the factor that was most significantly associated with presence of behavioral disturbance was the frequency of relapses per year (*P*=0.01). Though not statistically significant, poor socioeconomic status also showed a close association with behavioral disturbance (*P*=0.07). Sixty six percent of the children were receiving corticosteroids, but their use did not seem to contribute significantly to the development of behavioral disturbance. Age, gender or age of onset of illness did not play any significant role in determining the presence/absence of behavioral problems [Table 3].

**Table 3:** Association between sociodemographic and clinical variables and behavioral abnormality in nephrotic syndrome group

Variable	B	SE	Wald	df	Sig	Exp (B)
Age	0.561	0.427	1.728	1	0.189	1.753
Sex	−0.472	0.972	0.249	1	0.618	0.624
Age of onset	−0.510	0.551	0.859	1	0.354	0.600
Frequency of relapse	3.966	1.559	6.469	1	0.011	52.771
Steroid	−1.346	1.053	1.635	1	0.201	0.260
Low SES	−2.895	1.621	3.189	1	0.074	0.055

SES = Socioeconomic status

Another interesting finding was that, while all children in the matched group were school students, only 50% of nephrotic syndrome sufferers were going to school. This was despite the fact that the nephrotic syndrome group and control groups were comparable in terms of sociodemographic profile. The possible explanation and implications of these findings have been discussed subsequently.

## DISCUSSION

Any chronic illness is associated with the burden of psychosocial stress, something that is both brought on and aggravated by physical pain or disability. This is particularly true of illnesses that affect children, because of their innate vulnerability and the high emotional stakes involved for parents and other caregivers. It is therefore, natural to assume that behavior problems will be commoner in children with chronic disease, independent of the nature of the illness.

Epidemiological surveys suggest that at any given point of time, 6–12% of children are afflicted with serious chronic physical illness.[13] While some may adapt to this quite successfully, a significant number may not, resulting in social, psychological, and academic handicaps that may sometimes be more serious than the physical limitations of the underlying disease.

Studies addressing this issue have confirmed that behavioral problems such as neurosis, attention deficit, hyperactivity, misconduct in school, and adjustment problems are twice as common in children with chronic disease as in healthy children of the same age.[13]

Factors that seem to worsen a child's adjustment are: interferences with the daily functioning, normality of appearance, need for surgical procedures, and uncertain outcome. Ambiguous and uncertain conditions in a superficially normal-appearing child pose particular problems in adaptation.[14] Many of these stressors are present in children with nephrotic syndrome. Besides, prolonged corticosteroid treatment may contribute to behavioral disturbances in this especially vulnerable population.

In a study by Soliday *et al*., nearly 65% of children with steroid sensitive nephrotic syndrome having normal behavior at baseline showed anxious/depressive and/or aggressive behaviour during relapse, severe enough to be referred to a mental health provider. The children with abnormal behavior at baseline also showed worsening of their behavior during relapse. Regression analysis showed that prednisone dose was a strong predictor of abnormal behavior, especially increased aggression.[8]

A recent study from China reported that children with nephrotic syndrome had higher degrees of somatization, interpersonal sensitivity, depression, anxiety, hostility, fear, and paranoia compared to healthy controls (*P*<0.05). Besides, the parents of children with nephrotic syndrome showed increased introversion and neuroticism compared to controls.[9]

A study conducted in Northern India reported that a significant proportion of children with nephrotic syndrome show features of depressed, hyperactive, or aggressive behavior. Somatic complaints, social withdrawal, and poor school performance were also observed. The behavior abnormality scores correlated with the anxiety scores of the mothers of children with nephrotic syndrome.[7]

Other studies have suggested several areas of increased vulnerability among the parents and siblings of children with nephrotic syndrome.[15] The personality profiles of the siblings suggested decreased social confidence and a lesser degree of self-acceptance.[16]

A recent study evaluated health related quality of life (QOL) and psychosocial adjustment in patients with steroid-sensitive nephrotic syndrome. The QOL subscale “social functioning” was significantly impaired in children with nephrotic syndrome. Parents of the latter, however, rated four of a total of seven QOL subscales as abnormal. Psychosocial adjustment of patients was impaired at home and at school. Family climate, especially maternal distress, negatively affected both QOL and psychosocial adjustment in this group.[17]

Our study reveals that a significant proportion of children (68%) with nephrotic syndrome suffer from behavioral problems, compared to the age and sex matched control group. This prevalence is quite high and can possibly be explained by the fact that the study center (renal clinic of a state medical college) was a tertiary referral institute. Such high prevalence of behavioral disturbance is unlikely to be replicated in community studies. Another factor that must be kept in mind is the overrepresentation of lower socioeconomic status in the study sample. Nearly 84% of the patients belonged to lower socioeconomic status. It is likely that severe poverty in association with chronicity of disease contributed to the very high prevalence of behavior disturbance in the study sample. Subsequent analysis has, in fact, shown a close relation between poor socioeconomic status and behavioral abnormality in our sample.

The proportion of behavioral difficulties in the control group was estimated to be 21.6%. Previous estimates of behavior problems in Indian school children were around 14.6%, though these were probably underestimates, by the authors’ own admission.[18] In a WHO sponsored multicentric study in four developing countries including India, 12 to 29% of children attending primary health care facilities had identifiable psychiatric disorders.[19] In our sample of children with nephrotic syndrome, the commonest behavior abnormality noticed was hyperkinesis, followed by conduct disorder, learning disorder, and obsessive compulsive neurosis. This finding is in keeping with other studies of children with nephrotic syndrome.[7]

The factors that showed a significant association with behavior problem in the nephrotic syndrome children were frequency of relapse, followed by low socioeconomic status. Frequency of relapse is one of the most important indicators of disease course in nephrotic syndrome, as children having more than three relapses per year merit a separate diagnostic subcategory (“frequently relapsing type”). Increased frequency of relapse is associated with more frequent follow up visits at the clinic, resulting in more absenteeism from school, longer periods lost in illness, uncertainty and inactivity, isolation from peer groups, and inability to catch up with academic sessions. Frequent visits to the health care facilities also result in more work days lost for the caregivers, adding to the financial burden of the already economically compromised households. Thus, the burden of physical illness is further complicated by the associated socioeconomic difficulties that emerge when the child needs more intensive monitoring. This could possibly explain the significant association between frequency of relapse and behavioral maladjustment.

The association between low socioeconomic status and behavioral problems reinforce the assumption that chronic diseases produce difficulties in psychological adjustment not because of the disease process *per se*, but the serious social implications that they carry with them. The social burden is magnified by the presence of poverty.

Surprisingly, there was no significant association between corticosteroid treatment and behavior abnormality. However, the dose of corticosteroid received by most children in our study sample was moderate to low (1.5 mg/kg every alternate day). Studies that have reported significant correlation between corticosteroid use and behavior problems also noted that the observed behavioral changes occurred almost exclusively at the higher dose ranges of prednisone.[8]

Another important finding that emerged in our study was the association between presence of behavior disturbance and inability to attend school. Fifty percent of the children with nephrotic syndrome were out of school at a given point of time, a finding that has sinister implications. A similar study in India involving children with nephrotic syndrome also mentions lower school performance of these children compared to a control group.[7]

It is known that school presents particular challenges to the chronically ill children, both physically and socially. A school environment that includes verbal abuse and less peer support for ill children may be a problem. Teachers' insufficient knowledge about the illness and inability to spend adequate time with chronically ill children present barriers to the integration of the chronically ill child into the classroom situation.[20,21]

In the absence of adequate psychosocial intervention, many chronically ill children remain outside the scope of formal education, something that in turn increases their isolation and misery.

Increased frequency of relapse was associated with an increased prevalence of behavior disturbance which in turn predicted school dropout. A frequently relapsing course results in irregular attendance, and children may find it difficult to cope with the burden of missed lessons, interrupted peer interactions, and a punitive school environment. All these factors may contribute to the high rate of school dropouts in the nephrotic syndrome patients.
